# Enhanced vector surveillance to control arbovirus epidemics in Colombia

**DOI:** 10.26633/RPSP.2019.50

**Published:** 2019-06-07

**Authors:** Sarah Anne J Guagliardo, Susana Carolina Ardila Roldan, Liliana Santacoloma, Cesar Luna, Juan Manuel Cordovez Alvarez, Juan David Rojas Gacha, Mariana Mansur, Rebecca S Levine, Audrey Lenhart, Patricia Fuya Oviedo

**Affiliations:** 1 Centers for Disease Control and Prevention, Epidemic Intelligence Service, Atlanta Centers for Disease Control and Prevention Epidemic Intelligence Service, Atlanta Georgia United States of America Centers for Disease Control and Prevention, Epidemic Intelligence Service, Atlanta, Georgia, United States of America.; 2 Instituto Nacional de Salud, Grupo de Entomología Instituto Nacional de Salud, Grupo de Entomología Laboratorio Nacional de Referencia Bogotá Colombia Instituto Nacional de Salud, Grupo de Entomología, Laboratorio Nacional de Referencia, Bogotá, Colombia.; 3 Universidad de los Andes Universidad de los Andes Departamento de Ingeniería Biomédica Bogotá Colombia Universidad de los Andes, Departamento de Ingeniería Biomédica, Bogotá, Colombia.; 4 Departamento de Matemáticas, Universidad Nacional de Colombia Departamento de Matemáticas, Universidad Nacional de Colombia Bogotá Colombia Departamento de Matemáticas, Universidad Nacional de Colombia, Bogotá, Colombia.; 5 Task Force for Global Health, Decatur Task Force for Global Health, Decatur Georgia United States of America Task Force for Global Health, Decatur, Georgia, United States of America.; 6 Centers for Disease Control and Prevention, Division of Parasitic Diseases and Malaria, Atlanta Centers for Disease Control and Prevention, Division of Parasitic Diseases and Malaria, Atlanta Georgia United States of America Centers for Disease Control and Prevention, Division of Parasitic Diseases and Malaria, Atlanta, Georgia, United States of America.

**Keywords:** Mosquito vectors, epidemiological monitoring, vector control, Colombia, Mosquitos vectores, monitoreo epidemiológico, control de vectores, Colombia, Mosquitos vetores, monitoramento epidemiológico, controle de vetores, Colômbia

## Abstract

In the wake of the Zika epidemic, there has been intensified interest in the surveillance and control of the arbovirus vectors *Aedes aegypti* and *Aedes albopictus*, yet many existing surveillance systems could benefit from improvements. Vector control programs are often directed by national governments, but are carried out at the local level, resulting in the discounting of spatial heterogeneities in ecology and epidemiology. Furthermore, entomological and epidemiological data are often collected by separate governmental entities, which can slow vector control responses to outbreaks. Colombia has adopted several approaches to address these issues. First, a web-based, georeferenced *Aedes* surveillance system called SIVIEN AEDES was developed to allow field entomologists to record vector abundance and insecticide resistance data. Second, autocidal gravid oviposition (AGO) traps are deployed as an alternative way to measure vector abundance. Third, data collected by SIVIEN AEDES are used to develop mathematical models predicting *Ae. aegypti* abundance down to a city block, thus allowing public health authorities to target interventions to specific neighborhoods within cities. Finally, insecticide resistance is monitored through bioassays and molecular testing in 15 high-priority cities, providing a comprehensive basis to inform decisions about insecticide use in different regions. The next step will be to synchronize SIVIEN AEDES data together with epidemiological and climatic data to improve the understanding of the drivers of local variations in arbovirus transmission dynamics. By integrating these surveillance data, health authorities will be better equipped to develop tailored and timely solutions to control and prevent *Aedes*-borne arbovirus outbreaks.

## RESUMEN

Tras la epidemia del Zika, se ha intensificado el interés en vigilar y controlar los vectores de arbovirus *Aedes aegypti* y *Aedes albopictus*. Aun así, muchos de los sistemas existentes de vigilancia necesitan mejorar. En general son los gobiernos nacionales los que dirigen los programas de control de vectores, aunque estos programas se llevan a cabo a nivel local, por lo que no se tiene en cuenta la heterogeneidad del lugar en cuanto a las características ecológicas y epidemiológicas. Además, normalmente los datos entomológicos y epidemiológicos son recopilados por entidades gubernamentales distintas, lo que puede ralentizar el control de vectores durante un brote. Colombia ha puesto en marcha varias iniciativas para abordar estas cuestiones. La primera es un sistema en línea de geolocación del mosquito *Aedes,* llamado SIVIEN AEDES, para que los entomólogos de campo puedan registrar la abundancia de los mosquitos vectores y recoger datos sobre la resistencia a los insecticidas. La segunda es la implantación de ovitrampas autocidales para hembras grávidas (AGO, por su sigla en inglés), que son una manera alternativa de medir la abundancia de vectores. La tercera iniciativa es utilizar los datos recogidos por el sistema SIVIEN AEDES para elaborar modelos matemáticos que predigan la abundancia del *A. aegypti* hasta incluso en una cuadra de ciudad, de manera que las autoridades de salud pública puedan dirigir las intervenciones a vecindarios específicos dentro de las ciudades. Por último, Colombia está vigilando en quince ciudades prioritarias la resistencia a los insecticidas mediante ensayos biológicos y análisis moleculares, de esta forma se genera una base de datos exhaustiva sobre la que fundamentar las decisiones acerca del uso de insecticidas en las diferentes regiones. El paso siguiente será sincronizar los datos recopilados por el sistema SIVIEN AEDES con datos epidemiológicos y climáticos para poder entender mejor cómo se originan las variaciones locales en la dinámica de transmisión de los arbovirus. Al integrar estos datos de vigilancia, las autoridades sanitarias estarán mejor equipadas para encontrar soluciones oportunas y adecuadas para la situación específica, a fin de controlar y prevenir los brotes de arbovirus transmitidos por el *Aedes*.

## RESUMO

Depois da epidemia de zika, intensificou-se o interesse na vigilância e controle dos vetores arbovirais *Aedes aegypti* e *Aedes albopictus*, mas muitos dos sistemas de vigilância existentes poderiam ser aprimorados. Muitos programas de controle de vetores são dirigidos pelos governos nacionais, mas implementados no âmbito local, o que leva à desconsideração de heterogeneidades espaciais em aspectos ecológicos e epidemiológicos. Além disso, é comum que dados entomológicos e epidemiológicos sejam coletados por agências governamentais separadas, o que pode desacelerar o controle de vetores em resposta aos surtos. A Colômbia adotou vários enfoques para abordar esses problemas. Primeiro, um sistema de vigilância de *Aedes* georreferenciado e baseado na Internet, chamado SIVIEN AEDES, foi desenvolvido para permitir aos entomólogos de campo registrar a abundância de vetores e a resistência aos inseticidas. Segundo, ovitrampas letais para fêmeas grávidas estão sendo mobilizadas como maneira alternativa de medir a abundância vetorial. Terceiro, os dados coletados pelo SIVIEN AEDES estão sendo utilizados para desenvolver modelos matemáticos para prever a abundância do *Ae. aegypti* até o nível de quadra/quarteirão, permitindo assim às autoridades de saúde pública direcionar intervenções para bairros específicos em cada município. Finalmente, a resistência aos inseticidas é monitorada através de ensaios biológicos e testes moleculares em 15 cidades de alta prioridade, o que proporciona uma base abrangente para subsidiar decisões sobre o uso de inseticida em diferentes regiões. O próximo passo será sincronizar os dados do SIVIEN AEDES com dados epidemiológicos e climáticos para melhorar a compreensão dos fatores que impulsionam variações locais na dinâmica da transmissão arboviral. Ao integrar esses dados de vigilância, as autoridades de saúde estarão mais bem equipadas para desenvolver soluções personalizadas e oportunas para controlar e prevenir os surtos de arbovírus transmitidos por mosquitos do gênero *Aedes*.

The urban/peri-urban mosquitoes *Aedes aegypti* and *Aedes albopictus* are responsible for transmitting many arboviruses, including Zika, dengue, chikungunya, and yellow fever. Since the 2015 emergence of Zika in Brazil and associated cases of microcephaly (1), there has been intensified interest in mosquito surveillance and control. Growing concerns about potential emergent arboviruses, in combination with the continued geographic expansion of *Ae. aegypti* and *Ae. albopictus*, point to the need for increased investment in vector surveillance. Given the lack of effective chemotherapeutics and suitable vaccines for dengue, Zika, and chikungunya, arbovirus control programs rely heavily on the suppression of vector populations to prevent epidemics and control the spread of outbreaks.

Both *Ae. aegypti* and *Ae. albopictus* are invasive in the Americas. *Ae. aegypti* was introduced from Africa via trade ships in the seventeenth through nineteenth centuries, and *Ae. albopictus* was introduced more recently via the used tire trade in the 1970s–1980s (2, 3). In the mid-twentieth century, *Ae. aegypti* was nearly eradicated as a result of an aggressive Pan American Health Organization yellow fever control program, but the program was abandoned as yellow fever cases waned, resulting in the recovery of mosquito populations. Since that time, *Ae. aegypti* and *Ae. albopictus* populations have persisted in the Americas, with the former mosquito generally dominating in highly urbanized areas and the latter in peri-urban and suburban areas.

Today, vector control programs exploit the anthropophillic nature of these mosquitoes by eliminating artificial water-holding containers that may serve as potential larval habitats and by applying larvicides, residual insecticides, and space sprays of adulticides in and around homes. Although there is some evidence that these interventions can be effective, vector control programs can fall short in two important ways. First, vector control programs sometimes assume a top-down structure in which central governments dictate the interventions applied in counties or cities. As a result, important spatial heterogeneities in ecology, entomology, and epidemiology can be overlooked. Second, epidemiological and entomological data are traditionally collected independently by different governmental entities and are only later merged for analyses. This division between vector data and human data makes it difficult to respond quickly to an outbreak and to disentangle the relationships among mosquito population dynamics, vector control interventions, ecological conditions, and human cases of disease. Indeed, these relationships are currently not well characterized or understood (4), and, consequently, interventions are sometimes driven by political pressure rather than empirical evidence.

Here, we describe an initiative led by Colombia’s National Institute of Health (INS) that addresses the need to account for spatial heterogeneity in vector control programs, while simultaneously setting the stage for combining entomological and epidemiological data into the same system. We also report on a more comprehensive approach to monitoring insecticide resistance, which is a fundamental component of the architecture of arbovirus surveillance and control programs.

## ENHANCING VECTOR SURVEILLANCE IN COLOMBIA: AN INTEGRATED APPROACH

The Colombian National Entomology Network is a unit within the INS that is tasked with developing vector distribution maps, providing reference laboratory services, and compiling data on vector surveillance and control activities as reported by field entomologists from each of the country’s 32 departments. As of 2017, *Ae. aegypti* was found in all 32 departments of Colombia, and *Ae. albopictus* had been reported in 11 of the 32 (34%), with geographic distribution highly dependent on subdepartmental factors such as access to oviposition sites, human population density, temperature, and altitude below 2 200 m. Data exist at coarse scales, but detailed, fine-resolution information about these covariates, in addition to mosquito presence and abundance data, would allow public health authorities to more effectively tailor control activities to mitigate local risk factors. To address this discrepancy, in 2016 the INS collaborated with the U.S. Centers for Disease Control and Prevention (CDC) to develop plans to: 1) implement an enhanced entomological surveillance system for arbovirus vectors; 2) trial autocidal gravid oviposition traps (AGO traps) to improve measures of vector abundance; 3) model *Ae. aegypti* abundance using baseline entomological data to determine neighborhoods and city blocks at greatest risk; and 4) comprehensively monitor insecticide resistance trends in selected cities.

A web-based surveillance system for malaria vectors (Sistema de Información para la Vigilancia Entomológica (SIVIEN) MALARIA) is used by INS for compiling data and generating reports related to malaria transmission and control, yet no parallel system existed for *Ae. aegypti* or *Ae. albopictus*, despite their public health importance and widespread distribution in Colombia. A similar web-based system for arbovirus vectors, SIVIEN AEDES, has now been developed, and can combine vector data into a nationally accessible geographic information system (GIS) using the QGIS software program (http://qgis.osgeo.org). Field entomologists throughout Colombia can access SIVIEN AEDES to record information on *Aedes* insecticide resistance (described below) and abundance (measured by both immature and adult indices). They can also report the discovery of these species in new geographical areas. An associated mobile data collection app called *Aedes* INSpector allows SIVIEN AEDES to compile data in real time and facilitates the prompt generation of statistical reports. This is useful for rapidly detecting spatiotemporal changes in vector distribution as well as for monitoring and evaluating vector control programs.

*Aedes* surveillance commonly relies on measurements of immature mosquitoes, obtained either via container surveys or ovitraps. To determine whether improved information can be gained through novel measures of adult mosquito abundance, a pilot study using autocidal gravid oviposition traps (5) (AGO traps) was undertaken in five cities in Colombia. Data obtained from these traps are being compared with the data obtained via traditional immature surveys. Qualitative evaluations are also being carried out to assess the acceptability of AGO traps in households and to evaluate the ease of processing material collected from the traps.

When considered together with epidemiological information, entomological data can be used to guide when and how to mount an elevated vector control response to arbovirus outbreaks. In the same subset of five cities in which AGO traps were installed, mathematical models are being developed in the MATLAB computer program to predict *Ae. aegypti* abundance at each life stage. The models are adaptive in nature: data on the number of eggs, larvae, pupae, and adult mosquitoes are collected weekly and are continually input into differential equations to evaluate mosquito population dynamics. Results are plotted on heat maps, displaying the approximate number of mosquitoes per city block where the AGO traps were placed. Local authorities can then utilize this information and target additional vector control activities to the specific city blocks with the greatest abundance of mosquitoes. If successful, the INS will expand this approach to monitor mosquito populations in other areas of arbovirus risk.

Insecticide resistance in Colombia is of increasing concern, and data from 2004 through 2015 indicate increasing resistance to pyrethroids among *Ae. aegypti* populations. The main insecticides used in Colombia for arbovirus control include the organophosphates malathion and pirimiphos-methyl, and the pyrethroids deltamethrin and lambda-cyhalothrin. Although pyrethroid resistance has been linked to the *kdr* Il,016 substitution on the voltage-gated sodium channel gene on the north coast of Colombia (6), there is a need to characterize other mechanisms of resistance, as well as the intensity of resistance and its impact on the success of vector control interventions throughout the country. Accordingly, the intensity of resistance is being measured through bioassays in 15 cities, and these results are being compared with historical information on vector control interventions. Molecular testing is being conducted to determine the presence of additional *kdr* mutations (Leu410, Iso1016, and Cys1534), in addition to biochemical tests to determine metabolic mechanisms of resistance. To inform decision-making about which insecticides should be applied in different regions, maps of resistance patterns and their underlying mechanisms are being developed.

## CONCLUSION

Zika, dengue, chikungunya, and yellow fever are arboviruses of major concern in Colombia, and robust vector surveillance and control programs are necessary to prevent disease transmission. The enhanced surveillance activities we describe here provide a timely example of how entomological data can be collected through innovative digital platforms, with the aim of improving vector control decision-making at a local scale. Colombia’s INS is also planning to synchronize entomological data (collected through SIVIEN AEDES) and epidemiological data (collected through the country’s Public Health Surveillance System (SIVIGILA) (https://www.minsalud.gov.co/salud/Paginas/SIVIGILA.aspx)). Ultimately, combining epidemiological and entomological data will help elucidate patterns that both improve scientific understanding of local transmission dynamics and guide decisions about when and how to scale up interventions in response to outbreaks.

## Author contributions.

All the authors conceived the original ideas. JMCA and JDRG developed the mathematical models. SJG, AL, and RSL wrote the paper. All the authors reviewed and approved the final version.

## Funding.

This publication was made possible through support provided by the Office of Infectious Disease, Bureau for Global Health, U.S. Agency for International Development, under the terms of an Interagency Agreement with CDC. The opinions expressed herein are those of the authors and do not necessarily reflect the views of the U.S. Agency for International Development.

## Disclaimer.

The findings and conclusions in this paper are those of the authors and do not necessarily represent the official position of the Centers for Disease Control and Prevention. In addition, the authors hold sole responsibility for the views expressed in the manuscript, which may not necessarily reflect the opinion or policy of the *RPSP/PAJPH* or the Pan American Health Organization (PAHO).
